# Reviewing the Raters: A Study of Orthopedic Oncology Rating Outcomes During COVID-19

**DOI:** 10.7759/cureus.70829

**Published:** 2024-10-04

**Authors:** Megna Panchbhavi, Alexander Yu, Jie Chen, John C Hagedorn, Cory Janney, Daniel Jupiter

**Affiliations:** 1 Department of Applied Computational Statistics and Mathematics, University of Notre Dame, Indiana, USA; 2 School of Medicine, University of Texas Medical Branch, Galveston, USA; 3 Department of Orthopedic Surgery and Rehabilitation, University of Texas Medical Branch, Galveston, USA; 4 Department of Orthopedic Surgery, Naval Medical Center San Diego, San Diego, USA; 5 Department of Preventive Medicine and Community Health, University of Texas Medical Branch, Galveston, USA

**Keywords:** covid, oncology, orthopedic, outcomes, rating, telemedicine

## Abstract

Background

During the 2020 COVID outbreak, telemedicine usage rates skyrocketed. We investigated the effect of telemedicine availability and demographic factors on orthopedic oncologist ratings and review rates. We also investigated patient-reported positive and negative experiences on www.heathgrades.com to determine which physician interaction factors were more importantly perceived by patients.

Methods

All orthopedic oncologists (n = 141) on www.healthgrades.com were selected for review through October 2020. Information obtained included telemedicine availability, gender, age, location of practice, review rate (number of reviews per 1000 days), mean star rating, and patient-reported positive and negative physician interactions. Mean star ratings and number of ratings were compared with respect to demographic information and telemedicine availability. Mean star ratings and review rates of orthopedic oncologists offering telemedicine were compared before and after January 01, 2020. Patients perceived positive and negative physician interaction factors were reported as a frequency of pooled positive or negative interaction factors, respectively.

Results

There were no significant differences in mean star rating or review rate with respect to demographic factors, telemedicine availability, or telemedicine offering orthopedic oncologists before and after January 01, 2020. There were increased positive interactions of both physician independent and dependent factors and increased negative interactions of time dependent factors.

Conclusion

Patients reported an increased frequency of negatively perceived wait times and rushed appointments. Telemedicine availability has been previously cited as a cost-effective means of reducing wait times. Prospective investigations are needed to determine if telemedicine can be utilized as a cost-effective means of improving patient care and satisfaction.

## Introduction

Previous investigations have explored various physician ratings and review outcomes and reported multiple factors that are associated with higher and lower physician ratings [1-8]. Moreover, with increased accessibility to the internet, American patients are now increasingly participating and utilizing physician rating websites to select physicians [9,10]. Hanauer et al. previously reported that reviews from physician rating sites are likely to influence what physician a patient chooses, with 28.1% of patients willing to seek out a physician with a strong positive review and 27% of patients who would avoid a physician with a strong negative review [10]. Additionally, the onset of 2020 came with the novel SARS-CoV-2 virus, a positive single-stranded highly infectious RNA virus, resulting in a previously unprecedented worldwide pandemic with direct effects on access to healthcare [11]. Specifically in the United States (US), following various state-specific quarantine procedures, multiple institutions reduced face-to-face contacts to reduce the spread of the virus [12]. As a result of these novel circumstances, telemedicine became widely used as a complementary alternative to physician-patient appointments [11].

During this period, telemedicine usage rates skyrocketed, with the McKinsey COVID-19 consumer healthcare survey reporting a US consumer rate of 49% in 2020 by late April, in contrast to a mere 11% in 2019 [11]. As a result, understanding novel, modern interaction factors potentially affecting physician ratings in the new decade may result in improved patient ratings outcomes or perceived physician-patient relationships. While several physician specialties have examined the effect of various physician demographic factors with respect to rating outcomes and perceived physician-patient relationships [1-8], to our knowledge, no such study has been utilized for orthopedic oncologists; consequently, whether these factors affect orthopedic oncology ratings are unknown. Understanding the factors influencing physician ratings, particularly in the context of telemedicine, can help orthopedic oncologists and healthcare providers enhance patient satisfaction and improve the physician-patient relationship. This insight could contribute to better patient retention, improved healthcare access, and potentially more positive health outcomes, as patients who feel more satisfied with their interactions are more likely to adhere to treatment recommendations and follow-up care. We sought to perform a preliminary study into previously explored physician rating factors [1-8] that could potentially affect orthopedic oncologists’ ratings in addition to determining whether telemedicine utilization status could affect overall orthopedic oncologists’ ratings and review rates. Additionally, we investigate patient-reported positive and negative experiences on www.heathgrades.com to determine whether physician interactions factors were perceived more negatively or positively by patients.

## Materials and methods

All Doctor of Medicine (MD) or Osteopathic Medicine (DO) orthopedic oncologists practicing in the US and reported on www.healthgrades.com were selected for review. A total of 141 orthopedic oncologists were found. Orthopedic oncologist sex, age, practicing state, telemedicine availability, average ratings, number of reviews, earliest and most recent dates of reviews, and patient-reported positive and negative physician interaction factors were extracted for each practicing orthopedic oncologist through October 2020. Physician sex was reported as male or female. Physician age was reported in terms of one of the following categories: not reported, less than 40 years of age, 40-50 years of age, 50-60 years of age, 60-70 years of age, and over 70 years of age. Physician-practicing states were categorized into the following US regions: Midwestern, Southern, Northeastern, or Western regions. Average ratings were calculated for each orthopedic oncologist utilizing telehealth medicine before and after January 01, 2020. The review rate was calculated for each orthopedic oncologist before January 01, 2020, as a function of the number of ratings per 1000 days, where the number of days is a function of the earliest provided review date of all telemedicine offering orthopedic oncologists subtracted from January 01, 2020. The review rate was similarly calculated for each telemedicine-offering orthopedic oncologist after January 01, 2020, as a function of the number of ratings per 1000 days, where the number of days is a function of the most recent provided review date of all telemedicine-offering orthopedic oncologists subtracting January 01, 2020. The frequency of positive and negative physician interaction factors perceived by patients was pooled for all physicians and compared with regard to positive and negative factors (e.g., frequency of positive appointment wait times vs frequency of negative appointment wait times).

The Mann-Whitney U test was used to compare mean star ratings and review rates with respect to gender or telehealth medicine availability of orthopedic oncologists. The Kruskal-Wallis test was used to compare mean star ratings and mean number of ratings with respect to reported physician age category or reported US geographic region. The Wilcoxon rank-sum test were used to compare reported mean star ratings and number of ratings per 1000 days before and after January 01, 2020 in orthopedic oncologists offering telemedicine services. Statistical significance was defined at p-value < 0.05.

## Results

A total of 141 orthopedic oncologists were identified in 37 states covering the West, Midwest, South, and Northeast regions of the US on www.healthgrades.com. The distribution of all physician ratings (not shown) was left skewed and ranged between an average rating of 2 to 5 stars with a median rating of 4.5 stars and an average total rating of all orthopedic oncologists of 4.23 +/- 0.75 stars. The distribution of the number of ratings per physician was right-skewed with a median number of ratings per physician of 13 ratings and an average number of ratings per physician of 18.68 +/- 23.53. There were no significant differences in orthopedic oncologist mean rating or review rate with respect to sex, age category, or US relative geographic region (Tables 1-2). There were 27 orthopedic oncologists that offered telehealth medicine services. Comparison of these telemedicine offering orthopedic oncologists before and after January 01, 2020, with respect to mean rating and review rate yielded no significant differences (Tables 1-2).

**Table 1:** Average patient-reported ratings (out of 5 stars) of orthopedic oncologists The mean orthopedic oncologist patient-reported rating (out of 5 stars) on healthgrades.com with respect to sex, telemedicine availability, US region, age category, and comparison of telemedicine offering orthopedic oncologists before and after January 01, 2020

Category	No.	Mean rating	p-value
Sex			
Male	117	4.24	0.70
Female	24	4.22	
Telemedicine availability			
Yes	52	4.26	0.42
No	89	4.21	
US region			
Northeast	23	4.16	0.1742
Midwest	33	4.27	
South	54	4.4	
West	31	3.99	
Age (years)			
<40	11	4.26	0.64
40-50	43	4.32	
50-60	36	4.22	
60-70	26	4.18	
>70	7	3.66	
Orthopedic oncologists offering telemedicine before and after January 01, 2020			
Before January 01, 2020	27	4.32	0.46
After January 01, 2020	27	4.32	

**Table 2:** Average number of reviews per 1000 days of orthopedic oncologists The average number of patient-reported reviews per 1,000 days with respect to sex, telemedicine availability, US region, age category, and comparison of telemedicine offering orthopedic oncologists before and after January 01, 2020

Category	No.	Mean number of reviews (per 1,000 days)	p-value
Sex			
Male	117	19.51	0.27
Female	24	14.63	
Telemedicine availability			
Yes	52	21.08	0.16
No	89	17.32	
US region			
Northeast	23	18.88	0.97
Midwest	33	13.7	
South	54	18.54	
West	31	22.42	
Age (years)			
<40	11	11.55	0.65
40-50	43	18.07	
50-60	36	20.56	
60-70	26	19.58	
>70	7	13.29	
Orthopedic oncologists offering telemedicine before and after January 01, 2020			
Before January 01, 2020	27	10.53	0.39
After January 01, 2020	27	12.53	

Overall, there were more patient-reported positive orthopedic oncologist vs. negative orthopedic oncologist factors (12,413 positive vs. 2,448 negative) (Table 3). There was increased frequency of positive ratings for the following orthopedic oncologist interaction factors: orthopedic oncologist’s staff friendliness, explanation of conditions by the orthopedic oncologist, listening and answering questions, trusting provider decisions, ease of appointment scheduling, and facility comfortability. In contrast, there were increased frequency of negative ratings for the following orthopedic oncologist interaction factors: orthopedic oncologist appointment time and waiting time.

**Table 3:** Patient-reported frequencies of positively and negatively perceived characteristics of physician interactions The frequencies of patient-reported positive and negative orthopedic oncologist interaction factors

Physician interaction characteristics	Positive	Negative	Total
Appointment time	227	227	454
Waiting time	234	1031	1265
Staff friendliness	2057	177	2234
Physician explanation	1963	244	2207
Physician listening & answering questions	1891	235	2126
Trust in provider's decisions	1985	258	2243
Ease of appointment scheduling	1923	180	2103
Facility comfortability	2133	96	2229
Total	12413	2448	14861

## Discussion

Through analyzing ratings and reviews of all orthopedic oncologists on healthgrades.com, we report no significant differences with orthopedic oncologist ratings or the number of ratings with regard to sex, age, or US geographic region. To our knowledge, no such study has been performed on orthopedic oncologist ratings and review rates; however, these findings are concordant with previous investigations in specialties including radiation oncology, hand surgeons, spine surgeons, and urology with regard to physician ratings or review rates [4,5,7].

While the patient perception of orthopedic oncologist interactions resulted in a higher frequency of patient-reported positive interaction factors, not all factors were more positively reported. Further, of the factors that were more frequently reported as positive by patients, not all factors were physician-dependent, concordant with prior specialty-dependent studies [5,8,13-15]. We report two orthopedic oncologist-dependent interaction factors (physician explanations and listening/answering questions, trust in provider decisions) and three orthopedic oncologist-independent interaction factors (staff friendliness, ease of appointment scheduling, facility comfort) that were more frequently reported positive. Of the factors that were more frequently reported as negative by patients, factors were interestingly both physician-dependent and independent, discordant with prior specialty-dependent studies [5,8,13-15]. We report two time-dependent orthopedic oncologist interaction factors that were more frequently reported as negative by patients, physician appointment time, and waiting times. Interestingly, time-dependent physician interaction factors have been widely cited as a source of patient dissatisfaction. Velasco et al. investigated perceived patient factors affecting orthopedic foot and ankle surgeons and reported that a common factor mentioned in negative comments was waiting time [8]. Further, a German study into physician-rating websites also cited waiting time as a source of patient-reported dissatisfaction in addition to physician-independent factors such as interactions with office staff, wait times, and nonclinical practice characteristics, providing evidence that physician-independent and time-dependent factors dominate negatively perceived patient-physician interactions, possible even internationally [13]. Our findings suggest that patient perception of orthopedic oncologist interaction factors is variable and can be influenced by patient perception with resultant over-/underreporting of orthopedic oncologist interaction factors. Additionally, these results provide potential avenues for orthopedic oncologists in the US to improve patient satisfaction.

Interestingly, we report no significant differences with regard to average orthopedic oncologist rating and review rate between orthopedic oncologists offering telemedicine availability and those that do not. Moreover, of these physicians providing telemedicine availability, there were no significant differences before and after January 1, 2020, with respect to physician ratings or review rate, suggesting potentially comparable and complementary utility of telemedicine appointments to in-person appointments. Though no significant differences were reported with regard to telemedicine vs. nontelemedicine availability, there are limitations to consider from this study. It is possible that more time and responses are needed to adequately observe a significant difference with regard to orthopedic oncologist average rating or number of reviews. The threshold date of January 1, 2020, for telemedicine availability may be more stringent than actualized as COVID restrictions were determined by state and region-specific guidelines resulting in nonsimultaneous provider availabilities; subsequently, orthopedic oncologist telemedicine availability may have been more readily available at a later and different date per physician, with resultant different rating and review rate outcomes [16]. Nonetheless, these findings are interesting as they suggest that telemedicine appointments are a potentially novel physician interaction factor that may complement in-person appointments with comparable physician ratings and review rate outcomes to previous in-person appointments in orthopedic oncology.

As previously described, time-dependent factors are a widely cited source of patient-reported physician interaction dissatisfaction. Telemedicine offers the potential to reduce wait times and unnecessary appointments for patients who are seeking outpatient services. Kruse et al. previously performed a retrospective review of published telehealth program implementation in nonspecific specialties and its effect on patient satisfaction from 2010 to 2017 and reported that telehealth interventions decreased wait times in addition to missed appointments, reduced readmissions, and improved medication adherence [17-22]. Moreover, nonspecific specialty consensus of telemedicine effectiveness and efficiency factors reported improved outcomes in chronically ill patients’ ease of use. It decreased costs and travel times and improved communication [17-22]. A separate systematic review more specifically studied the effect of telemedicine on subcategorized specialties and diseases and found telemedicine as a cost-effective tool specifically in delivering outpatient pulmonary care to rural populations, diabetic retinopathy, physical activity and diet counseling, eating disorders, and psychotherapy for depression [23-27]. Specifically, the telemedicine cost-effectiveness for these specialties revolved around utilization in outpatient appointments, decreased time and travel costs, and increased quality of life years and outcomes [23-27]. Critically, this study noted that different specific specialty diseases such as those of dermatology, asthma, and lung cancer were not cost-effective, secondary to increased burden of costs on the dermatological provider’s end in addition to no significant differences in in-person vs. telemedicine costs of pulmonary diseases [23]. Nonetheless, these results suggest telemedicine as a potential solution for increased negatively perceived orthopedic oncologist wait times. However, its effectiveness, cost optimization, and patient outcomes concerning orthopedic oncology need to be further evaluated.

This investigation encountered multiple restrictions. The reviewed orthopedic oncologists on www.healthgrades.com may not represent the general population. Additionally, not all reviewed orthopedic oncologists disclosed public information such as gender, age, or location on their healthgrades.com profile. Further, the review of one physician-rating website, as opposed to the review of multiple physician-rating websites, could be a limitation secondary to lost physicians or at risk to selection bias. Because this study was performed in a retrospective manner, higher level evidence such as prospective or randomized controlled trials are needed determine the effect of telemedicine on orthopedic oncologist average ratings or number of ratings. Nonetheless, our findings were comparable previous investigations in other specialties; additionally, various websites included double reporting of physicians. Further, physician characteristics are usually part of a structured view across multiple websites and include physician availability, staff characteristics, facility/location, bedside manner, time with doctor, technical aspects of care, and communication/education, suggesting lower selection bias [13]. Prior specialty-dependent investigations on physician rating outcomes performed a selective randomization of physicians based on specialties, while our study captured all orthopedic oncologists reported on www.healthgrades.com, thus lowering risk of selection bias compared to previous investigations [1-8]. Finally, we performed a novel investigation into telemedicine availability in light of novel 2020 events and orthopedic oncologist rating outcomes in addition to patient perception of physician characteristics of orthopedic oncologists, describing potentially new sources of patient dissatisfaction that may be addressed and further investigated in other physician specialties.

## Conclusions

Orthopedic oncologist ratings and review rates were not influenced by demographic data. Interestingly, patients reported an increased frequency of negatively perceived wait times and rushed appointments. Although telemedicine availability did not influence orthopedic oncologist ratings or the number of reviews, further investigations into perceived patient wait time and appointment times are needed to determine if telemedicine can be utilized as a cost-effective means of improving patient care and satisfaction.

Despite the expected nature of some results, the findings provide a framework for orthopedic oncologists to enhance both patient-provider communication and clinic operations, improving patient satisfaction and care outcomes. Addressing negative aspects like waiting times and appointment duration can lead to more efficient practices and potentially reduce patient anxiety in the oncology setting.
